# A genome-wide association analysis identifies *PDE1A*|*DNAJC10* locus on chromosome 2 associated with idiopathic pulmonary arterial hypertension in a Japanese population

**DOI:** 10.18632/oncotarget.20459

**Published:** 2017-08-24

**Authors:** Mai Kimura, Yuichi Tamura, Christophe Guignabert, Makoto Takei, Kenjiro Kosaki, Nobuhiro Tanabe, Koichiro Tatsumi, Tsutomu Saji, Toru Satoh, Masaharu Kataoka, Shigeo Kamitsuji, Naoyuki Kamatani, Raphaël Thuillet, Ly Tu, Marc Humbert, Keiichi Fukuda, Motoaki Sano

**Affiliations:** ^1^ Department of Cardiology, Keio University School of Medicine, Tokyo, Japan; ^2^ Univ Paris-Sud, Université Paris-Saclay, Le Kremlin-Bicêtre, France; ^3^ AP-HP, Service de Pneumologie, Hôpital Bicêtre, Le Kremlin-Bicêtre, France; ^4^ Inserm UMR_S 999, Hôpital Marie Lannelongue, Le Plessis-Robinson, France; ^5^ Department of Cardiology, International University of Health and Welfare Mita Hospital, Tokyo, Japan; ^6^ Center for Medical Genetics, Keio University School of Medicine, Tokyo, Japan; ^7^ Department of Respirology, Graduate School of Medicine, Chiba University, Chiba, Japan; ^8^ Department of Advanced Medicine in Pulmonary Hypertension, Graduate School of Medicine, Chiba University, Chiba, Japan; ^9^ Department of Pediatrics, Toho University, Medical Center, Omori Hospital, Tokyo, Japan; ^10^ Department of Cardiology, Kyorin University School of Medicine, Tokyo, Japan; ^11^ StaGen Co. Ltd., Tokyo, Japan

**Keywords:** pulmonary arterial hypertension, genome-wide association study, novel therapeutic target

## Abstract

Pulmonary arterial hypertension (PAH) is a lethal disease that often affects the young. Although *Bone Morphogenetic Protein Receptor Type 2* gene (*BMPR2)* mutations are related with idiopathic and heritable PAH, the low penetrance and variable expressivity in PAH suggest the existence of other genetic and/or environmental factors. In this study, we aimed to identify novel genetic factors associated with PAH, irrespective of *BMPR2* mutation.

We performed genome-wide association study (GWAS) in a Japanese population comprising 44 individuals with idiopathic and heritable PAH, and 2,993 controls.

Seven loci identified in the genome-wide study were submitted to the validation study, and a novel susceptibility locus, *PDE1A*|*DNAJC10*, was identified that maps to 2q32.1 (rs71427857, P = 7.9 × 10^-9^, odds ratio in the validation study = 5.18; 95% CI 1.86 – 14.42). We also found the augmentation of PDE1A protein in distal remodeled pulmonary artery walls in idiopathic PAH patients.

Given that phosphodiesterase 5 inhibitors are effective for the treatment of idiopathic and heritable PAH, our findings suggest that PDE1A could be a novel therapeutic target of PAH.

## INTRODUCTION

Pulmonary arterial hypertension (PAH) is a rare but fatal disease, with an estimated mean survival period in untreated patients of approximately 3 years [1]. Pulmonary vascular remodeling, occurring mostly in the small to mid-sized pulmonary arterioles (≤ 500μm), is a hallmark of PAH. This process is ascribed to the increased proliferation, migration and survival of pulmonary vascular cells within the pulmonary artery wall, i.e. pulmonary arterial smooth muscle cells (PA-SMCs), endothelial cells, myofibroblasts and pericytes [2]. However, the etiology and pathogenesis of PAH remain obscure.

Although many familial cases of heritable PAH exhibit an autosomal dominant mode of inheritance, with the majority having mutations in the *Bone Morphogenetic Protein Receptor, Type 2* gene *(BMPR2)*, [3] the penetrance of *BMPR2* pathogenic variants is low and estimated to be 14% for males and 42% for females [4]. This low PAH penetrance in *BMPR2* mutation carriers likely results from a combination of currently unknown genetic, environmental, and lifestyle factors [5].

Germain and colleagues have recently published the results of a genome-wide association study (GWAS) performed in patients with idiopathic PAH. They reported that the *CBLN2* locus, which maps to 18q22.3, is a susceptibility locus for PAH, [6] however they excluded PAH patients carrying *BMPR2* mutations from their study. Because additional common genetic variations may influence PAH development in patients either with or without a *BMPR2* mutation, we decided to include idiopathic and heritable PAH (I/HPAH) patients carrying *BMPR2* mutations in our GWAS.

Our objective was to identify new genetic variants that confer a predisposition to I/HPAH using a GWAS, and to validate this association in a second independent internal cohort of I/HPAH patients.

## RESULTS

### Discovery of novel PAH candidate genes

For discovery study, we genotyped 23 PAH patients (genotyped on the Illumina Human Omni2.5-8 Beadchip ver 1.1) and compared the results to genotypes from 2,002 healthy controls (JPDSC-Phase 2). Table 1 shows the clinical characteristics of the I/HPAH patients in the discovery study. Of the original 2,006 healthy controls, four subjects were excluded; two subjects because of first-degree relatives, and two subjects because they showed high-degree heterozygosities. We had planned to exclude samples that were judged to be outliers by PCA analysis using Eigenstrat software, [7] as well as samples with >1% missing genotypes, however such samples were not observed.

**Table 1:** Patients’ genetic and hemodynamic characteristics in the discovery cohort

ID	Age	Sex	Ethnics	BMPR2 mutation	mPAP [mmHg]	CO [l/min]	PVR [dynes ▪ sec ▪ cm^-5^]
1	63	Male	Japanese	+	42	2.72	1088
2	56	Female	Japanese	+	41	2.70	1064
3	66	Male	Japanese	+	54	1.96	2208
4	59	Female	Japanese	-	54	4.40	856
5	30	Female	Japanese	-	58	2.71	1563
6	31	Male	Japanese	-	61	NA	NA
7	15	Female	Japanese	-	75	2.38	2320
8	59	Female	Japanese	+	56	3.90	1008
9	48	Female	Japanese	-	36	4.90	480
10	23	Female	Japanese	-	70	2.90	1656
11	27	Female	Japanese	-	74	1.86	2840
12	48	Female	Japanese	-	45	3.27	1101
13	48	Female	Japanese	-	60	NA	2408
14	40	Female	Japanese	+	57	1.93	2152
15	53	Male	Japanese	+	66	3.20	NA
16	48	Female	Japanese	-	50	2.03	1728
17	62	Female	Japanese	-	49	2.90	1200
18	35	Female	Japanese	-	35	4.50	488
19	31	Female	Japanese	-	42	4.32	593
20	22	Male	Japanese	+	85	1.85	3072
21	57	Female	Japanese	-	86	1.63	3840
22	46	Female	Japanese	-	49	6.39	330
23	17	Male	Japanese	+	83	5.90	1032

The hemodynamic data were evaluated in their initial right heart catheterization.

mPAP: mean pulmonary arterial pressure; CO: cardiac output; PVR: pulmonary vascular resistance; NA: not available.

**Figure 1:**
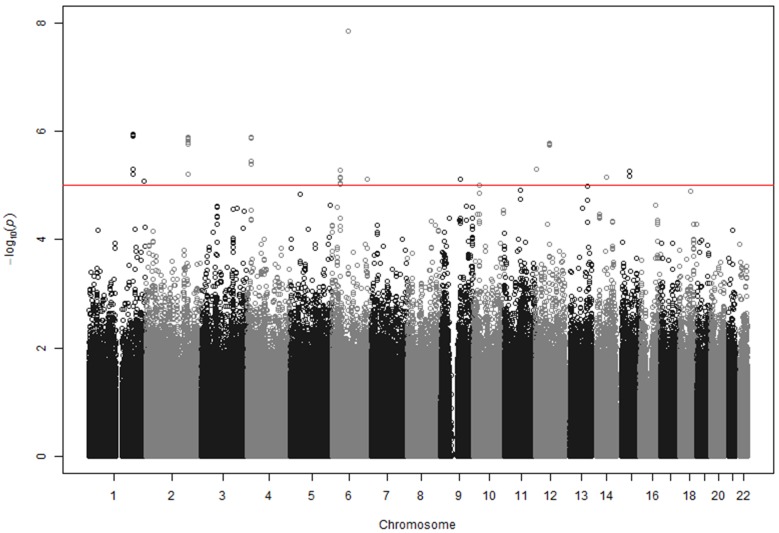
GWAS results at 1,347,690 SNPs with 23 cases and 2,002 controls under an additive model SNPs above red line showed the P values < 10^-5^. Eight regions contained more than one SNP with P values < 10^-5^. The figure was drawn using the R environment (R Version 2.15.3).

To perform GWAS analyses, 1,347,690 SNPs were selected because they showed minor allele frequencies (MAF) of > 0.01 in controls, a Hardy-Weinberg equilibrium (HWE) P-value of > 0.001 in controls, and a SNP call rate of > 0.95. Furthermore, these SNPs were included in both platforms (ver 1.0 and 1.1). To exclude potential stratification of the study population, we performed QQ-plot analysis and calculated the genomic inflation factor (λ) [8]. Neither the QQ-plot of log P-values shown in Supplementary Figure 1, nor the genomic inflation factor (λ) of 0.927 indicate the presence of any biases related to population stratification. Under an additive model for the effect of the minor allele at each SNP, we identified seven loci, each of which had more than one SNP with P values less than 10^-5^ (Figure 1, Table 2). We did not select loci that had a single SNP with a low P-value, because they often result from errors. The seven loci selected were *CDC73*|*KCNT2* on chr 1, *PDE1A*|*DNAJC10* on chr 2, *FAM184B* on chr 4, *MTCHI*|*FGD2* on chr 6, *TLE4*|*TLE1* on chr 9, *USP15* on chr 12 and *AQP9*|*LIPC* on chr 15. In Table 1, the SNP with the lowest P value for each locus is shown, although two SNPs are shown for the *PDE1A*|*DNAJC10* locus, which is considered especially important. As will be discussed later, PDE5 inhibitors are known to be effective for IPAH, and *PDE1A* codes for a phosphodiesterase (PDE) that is within the same PDE superfamily as PDE5 [14].

**Table 2:** 9 SNPs at 8 loci, each of which had more than one SNP with low (< 10^-5^) P values, in the discovery GWAS, and the results of the validation study

Stage^a^	rsID	CHR	POS	Gene	Minor allele	Major allele	MAF^b^	HWE^c^	Odds ratio	P-value
Discovery	rs12402555	1	194053792	*CDC73|KCNT2*	A	G	0.044	0.038	5.864	1.120E-06
Validation	rs12402555	1	194053792		T	C	0.057	0.766	0.883	8.714E-01
Meta analysis									4.142	1.492E-05
Discovery	rs71427857	2	183497840	*PDE1A|DNAJC10*	A	C	0.042	0.108	6.078	1.289E-06
Validation	rs71427857	2	183497840		A	C	0.046	1.000	5.181	1.636E-03
Meta analysis									5.759	7.927E-09
Discovery	rs13023449	2	183499313	*PDE1A|DNAJC10*	G	A	0.042	0.111	6.071	1.320E-06
Validation	rs13023449	2	183499313		G	A	0.046	1.000	5.163	1.674E-03
Meta analysis									5.748	8.301E-09
Discovery	rs11734719	4	17713542	*FAM184B*	G	A	0.168	0.814	4.657	1.264E-06
Validation	rs11734719	4	17713542		C	T	0.187	1.000	0.669	4.024E-01
Meta analysis									2.580	3.444E-04
Discovery	rs16868761	6	36955643	*MTCH1|FGD2*	A	G	0.010	1.000	12.310	5.134E-06
Validation	rs16868761	6	36955643		T	C	0.011	1.000	0.000	9.971E-01
Meta analysis									NA	NA
Discovery	rs11138921	9	83547081	*TLE4|TLE1*	G	A	0.243	0.720	4.179	7.802E-06
Validation	rs11138921	9	83547081		G	A	0.238	1.000	1.385	3.622E-01
Meta analysis									2.559	8.152E-05
Discovery	rs7316131	12	62669425	*USP15*	A	G	0.015	0.417	10.810	1.395E-06
Validation	rs7316131	12	62669425		A	G	0.016	1.000	0.000	9.965E-01
Meta analysis									NA	NA
Discovery	rs74886773	15	58638467	*AQP9|LIPC*	A	G	0.028	0.414	8.874	5.524E-06
Validation	rs74886773	15	58638467		A	G	0.034	0.312	0.000	9.947E-01
Meta analysis									NA	NA

Nine SNPs at seven loci identified by a discovery GWAS were validated by an independent study, followed by the calculation of P-value as a meta-analysis. MAF: minor allele frequency in the control group in the discovery phase (Phase 2). HME: P-value for the test of Hardy-Weinberg’s equilibirum (HWE) by the exact test.

A previous study reported that the *CBLN2* locus at ch 18q22.3, was associated with PAH [6]. We attempted to replicate these results with our GWAS. Although SNP rs2217560 (Position 70150939 on chr 18 in build 37) which was reported to be associated with PAH by Germain and colleagues, was not included in our platform, proximal SNPs were examined. However, we did not find any association between the 34 SNPs that were located close to rs2217560 (from Position 70121268 to 70181319) with PAH in our study (P > 0.15). Thus, our data did not successfully replicate the previously described association.

### Replication and validation of our GWAS study using another independent cohort

We next validated the associations observed in our discovery phase using another independent cohort of 21 patients and 991 controls (JPDSC-Phase 1). Table 3 shows the clinical characteristics of I/HPAH patients in the validation study. In this validation study, we examined the association of eight SNPs at seven loci (Table 2) with PAH using 21 cases and 991 controls (Table 1). The statistical method used to evaluate the association was identical to the one used for our first genome-wide study. Only rs71427857 and rs13023449, both at the *PDE1A*|*DNAJC10* locus, showed significant associations in the validation study (P=1.64 × 10^-3^ and P=1.67 × 10^-3^, respectively) (Table 2). The P-value threshold for each of the eight SNPs in the validation study was 0.0056 (0.05/9), in accordance with Bonferroni’s correction method for multiple comparisons.

**Table 3:** Patients’ genetic and hemodynamic characteristics in the replication cohort

ID	Age	Sex	Ethnics	*BMPR2* mutation	mPAP [mmHg]	CO [l/min]	PVR **[dynes ▪ sec ▪ cm**^**-5**^]
1	27	Female	Japanese	-	69	4.69	1176
2	41	Female	Japanese	-	74	3.40	1552
3	45	Female	Japanese	-	72	2.00	2584
4	43	Female	Japanese	-	82	2.20	2560
5	30	Female	Japanese	-	59	3.81	1008
6	45	Female	Japanese	-	42	3.50	824
7	59	Female	Japanese	-	46	2.60	1200
8	36	Female	Japanese	+	66	1.95	2378
9	39	Female	Japanese	-	49	3.10	1056
10	66	Female	Japanese	-	32	2.84	734
11	47	Female	Japanese	-	51	3.87	952
12	53	Female	Japanese	+	43	5.23	581
13	47	Female	Japanese	-	51	2.52	1460
14	42	Female	Japanese	-	39	4.50	622
15	53	Female	Japanese	-	47	6.38	451
16	45	Female	Japanese	-	54	3.62	1016
17	70	Female	Japanese	-	45	4.17	614
18	60	Female	Japanese	-	38	3.98	643
19	64	Female	Japanese	+	35	3.87	599
20	33	Female	Japanese	-	54	4.93	698
21	49	Female	Japanese	-	66	3.50	1412

The hemodynamic data were evaluated in their initial right heart catheterization.

mPAP: mean pulmonary arterial pressure; CO: cardiac output; PVR: pulmonary vascular resistance.

### Meta-analysis and imputation study

Meta-analyses using data from the discovery and validation studies indicated that P_meta_ for rs71427857 and rs13023449 were 7.93 × 10^-9^ and 8.30 × 10^-9^, respectively (Table 2). Both of these P_meta_ values are lower than the required threshold of 5 x 10^-8^, and were considered to be significant as a result of the meta-analysis. Odds ratios obtained from the validation study were 5.18 (95%CI 1.86 – 14.42) and 5.16 (95%CI 1.86 – 14.37) for rs71427857 and rs13023449, respectively. Since odds ratios obtained in the discovery phase are often overestimated, we consider values from the validation study more reliable.

**Figure 2:**
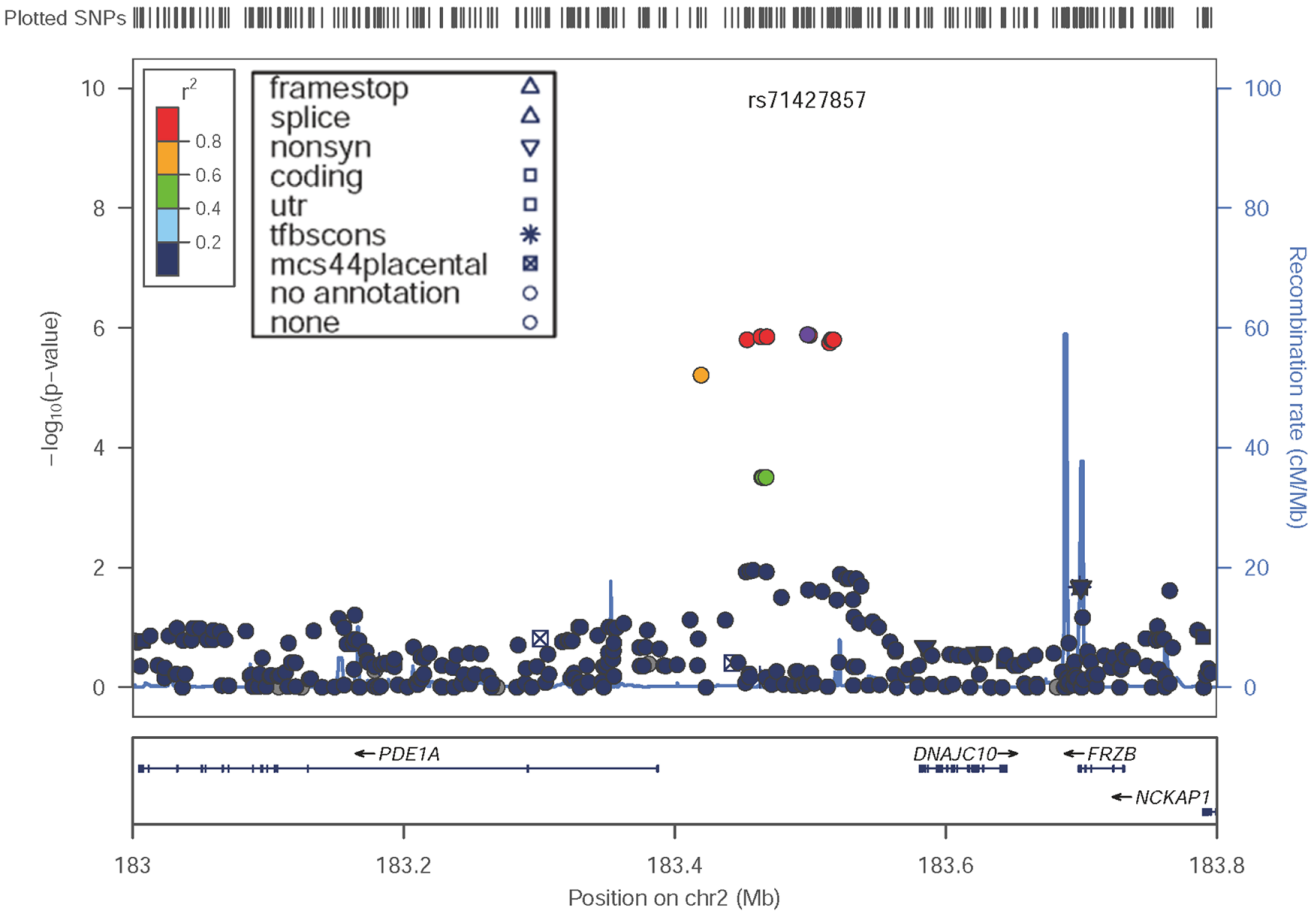
Locus-specific plots at the *PDE1A|DNAJC10* locus in the discovery GWAS The figure was drawn by LocusZoom Version 1.1^22^.

In our present study, regional plots near the *PDE1A*|*DNAJC10* locus indicated that the associated SNPs are clustered in the 5’ region of *PDE1A* (Figure 2).

We imputed genotypes for HapMap3 SNPs using IMPUTE version 2 (9) across the *PDE1A*|*DNAJC10* locus (Supplementary Figure 2). In general, the imputation results were consistent with the genotyped SNP results, although some ungenotyped SNPs in the same region were added as associated SNPs. To exclude the possibility of genotyping mutations that were in fact false positives, which had arisen by calling errors from SNP chips, we performed direct genotyping by Sanger sequence. All cases were directly sequenceded at the rs71427857 and rs13023449 locus. As shown by the mutant sample in Supplementary Figure 3, all cases with minor alleles showed the same results, both at rs71427857 and rs13023449 by the direct sequence method.

### Immunohistochemical studies and quantitative RT-PCR

**Figure 3:**
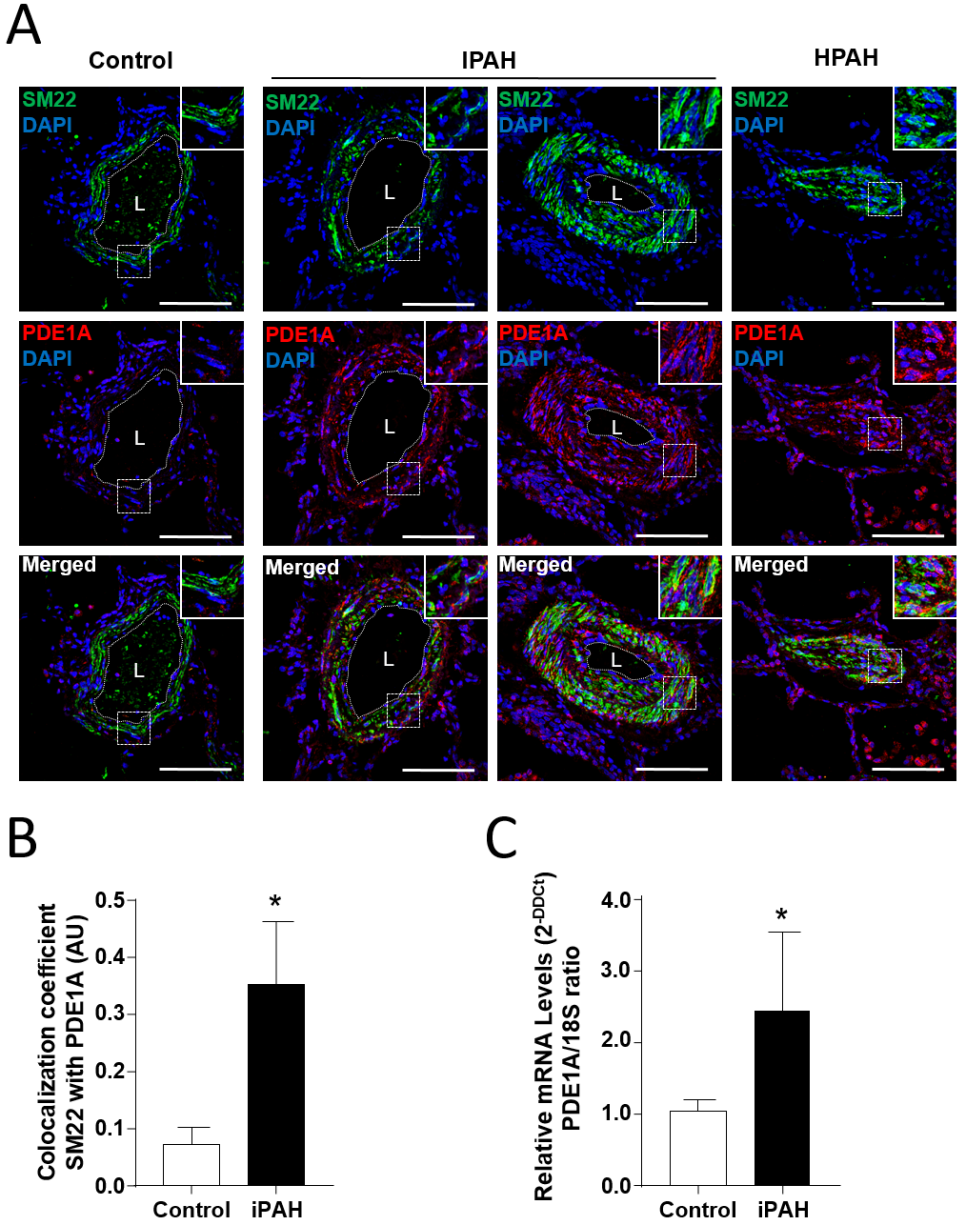
The augmentation of PDE1A expressions in pulmonary arterial smooth muscle cells (PA-SMCs) from patients with I/HPAH **(A)** Representative photomicrograph of PDE1A immunostaining in distal pulmonary arteries in lung sections of controls and idiopathic and heritable pulmonary arterial hypertension (I/HPAH) patients. Scale bars= 50 μm in all sections. L=vessel lumens. **(B)** Ratio of colocalization cells of PDE1A and SM22 positive cells in distal pulmonary arteries in lung sections from controls and IPAH patients. **(C)** PDE1A mRNA levels detected by quantitative reverse transcription PCR (RT-qPCR) in cultured PA-SMCs from controls and IPAH patients. * p-value < 0.05.

To confirm the contribution of the *PDE1A* gene to the pathogenesis of PAH, confocal microscopic analyses and double labeling with PDE1A and SM22 alpha, a marker of adult smooth muscle, were used to investigate the expression of PDE1A protein in lung specimens from 5 patients with PAH, and control subjects. We found strong staining of PDE1A in paraffin embedded lung tissue from patients with I/HPAH when compared to control subjects (Figure 3A). Interestingly, more intense immunoreactivity was noted for PDE1A protein in distal remodeled pulmonary artery walls from IPAH patients *versus* controls (Figure 3B). Also, the mRNA expression levels of PDE1A in PA-SMCs from IPAH were significantly higher than those from controls (Figure 3C), suggesting that the increased expression of PDE1A in PA-SMCs from the patients with IPAH was associated with the transcriptional activation.

## DISCUSSION

In this study, we performed GWAS in a Japanese population comprising 44 individuals with I/HPAH, and 2,993 controls. Out of seven loci identified in the discovery study, a novel susceptibility locus, *PDE1A*|*DNAJC10*, was identified in the replication study. More intense immunochemical staining for PDE1A protein in distal remodeled pulmonary artery walls was observed in PAH patients than controls.

Cyclic nucleotide PDEs play important roles in signal transduction by regulating intracellular cyclic nucleotide concentrations through the hydrolysis of cAMP and/or cGMP to their respective nucleoside monophosphates [9]. There are at least 11 families of one or more genes that encode PDE superfamily members. Furthermore, different proteins are synthesized from a single gene by different modifications of the encoding mRNA [14]. Members of the PDE1 family, such as *PDE1A*, are Ca^2+^/calmodulin-dependent PDEs that are activated by calmodulin in the presence of Ca^2+^ [10, 11].

PDEs have attracted attention from researchers, as well as pharmaceutical companies, because cAMP and cGMP are important signaling molecules in many tissues and organs [9]. Inhibitors of PDEs are likely to have a variety of pharmacological effects, many of which may be of clinical use. Nevertheless, at present, only a few PDE inhibitors are in widespread clinical use. For example, the PDE3 inhibitors, milrinone and cilostazol, are used to treat cardiovascular disease, [12, 13] and the PDE5 inhibitors, sildenafil, vardenafil and tadalafil, are useful for treating male erectile dysfunction [9]. PDE5 inhibitors are also useful for treating patients with pulmonary arterial hypertension [14].

Increase in the expression of PDE1A, as well as PDE5, has been shown in pulmonary arterial smooth muscle cells from patients with PAH [15]. In addition, Selective inhibition of PDE1 is known to augment the vasodilatory effect of inhaled nitric oxide in a PH model. Therefore, it is suggested that PDE1A is related with the development of PAH [16].

Genetic studies in human diseases may lead to an understanding of a protein’s function in the human body. For example, mutations in *PDE6A* and *PDE6B* are associated with autosomal recessive retinitis pigmentosa [17, 18]. Similarly, human *PDE4D* haplotypes and single-nucleotide polymorphisms (SNPs) correlate with ischemic stroke, and *PDE4B* SNPs are associated with schizophrenia [19, 20].

This study had several limitations. First, the number of I/HPAH patients included in this study was relatively small. One of the reasons why we could identify a novel susceptibility locus is that we used data of a large number of healthy controls. And since the ethnics of the patients were all Japanese, they were ethnically matched with the controls. In a previous GWAS study of Japanese population which identified genes associated with allopurinol-related Stevens-Johnson syndrome and toxic epidermal necrolysis, the identification was enabled by comparing only 14 patients with 991 healthy controls [21]. Second, we included not only young patients but also older populations both in discovery cohort and replication cohort suggesting that there is a possibility to include atypical I/HPAH patients among the cohort. However, all of the patients included in this study were diagnosed in experienced pulmonary hypertension centers, and the fact certifies all of the cases obtained typical characteristics of I/HPAH independent from the effect from other diseases such as left ventricular dysfunctions or pulmonary diseases regardless of the older ages. Third, we screened all I/HPAH cases for *BMPR2* mutations with Sanger sequencing. However, Sanger sequencing is not enough to identify patients with large deletions or duplications, and combination use of multiplex ligation-dependent prove amplication with Sanger sequencing might be more appropriate. Fourth, our data did not successfully replicate the CBLN2 finding in the previous study [6]. The different result might come from the difference of races included in each study; the genetic factors which modify PAH disease expression could be different between races. Further analysis is required to confirm the association of the locus and development of I/HPAH in it other cohorts with different races.

In conclusion, this GWAS in I/HPAH patients, irrespective of the presence of a *BMPR2* mutation, provided additional information about the association between a genetic locus, *PDE1A|DNAJC10*, and the disease, suggesting that PDE1A could be a novel therapeutic target of PAH.

## MATERIALS AND METHODS

### Study populations

We included patients with I/HPAH in this study. Diagnosis of PAH was hemodynamically confirmed for all cases included in the study by right-heart catheterization (discovery and validation stages). Cases were identified at three major pulmonary hypertension centers: Keio University, Chiba University, and Kyorin University in Japan. For all cases, PAH was defined as a mean pulmonary arterial pressure not lower than 25 mm Hg, and pulmonary capillary wedge pressure not higher than 15 mm Hg at baseline, according to protocols previously described [22]. A diagnosis of I/HPAH was declared only after clinical and biological investigations had eliminated all known causes of PAH. We excluded cases with complications such as lung or liver disease, even if not severe. After a confirmed diagnosis of I/HPAH, all cases were screened for *BMPR2* mutations by Sanger sequencing. Eight of 23 patients included in the discovery GWAS had mutations in the *BMPR2* gene, while 3 of the 21 patients included in the validation study had *BMPR2* mutations. We included only a single case from a family for the present study.

Controls consisted of 2,002 (Phase 2) and 991 (Phase 1) healthy Japanese individuals. Control samples were collected at two different times by the Japan PGx Data Science Consortium (JPDSC) [23]. Written informed consent was provided for enrollment in the study, and the GWAS study was approved by IRB of Keio University.

### GWAS and validation genotyping

Genomic DNA was isolated from whole blood and applied to the Illumina Human Omni2.5-8 Beadchip ver 1.1 SNP chips (cases) or Illumina Human Omni2.5-8 Beadchip ver 1.0 (controls Phase 2) SNP arrays. Genotyping conditions were as described in the manufacturer’s manual.

Validation genotyping was carried out at Keio University by Sanger sequencing of PCR products generated with forward primer 5’- GCAATGCTGCTTTGTTTCTCTG -3’ and reverse primer 5’ - TGACTGAGACTAGTGGGGAGTC-3’. The appropriate temperature for successful resolution were determined by the dHPLC melting algorithm. Samples with an altered dHPLC profile were sequenced using the BigDye Terminator cycle sequencing kit (Applied Biosystems, Foster city, CA) on an ABI 3730xl DNA sequencer (Applied Biosystems). The resulting sequences were compared with the reference sequence of the SNPs sites with the ABI SeqScape software (Applied Biosystems).

### Immunostaining

Lung specimens were fixed in 4% paraformaldehyde and embedded in Paraffin. Sections (5-μm) were dewaxed and progressively rehydrated. Lung sections were then blocked in 5% bovine serum albumin for 30 minutes at room temperature and stained with anti-PDE1A (Novus Biologicals, Lilles, France) and anti-SM22 (Santa Cruz Biotechnology, Heidelberg, Germany) antibodies overnight at 4°C. Secondary antibodies, conjugated with a fluorescent label (Interchim, Montluçon, France), were applied for 1 hour at room temperature. Nuclei were labelled using DAPI (Life technologies). Sections were mounted using ProLong Gold antifade reagent (Life technologies). Images were taken using a LSM700 confocal microscope (Zeiss). Overlap between the PDE1A and SM22 proteins, as represented by the Manders coefficient, was determined using the ZEN software (Blue edition, version 2012, Zeiss) from a minimum of 8 distal pulmonary arteries for each group.

### Quantitative RT-PCR

Total RNA was isolated from cultured PA-SMCs by RNeasy mini kit (Qiagen, Courtaboeuf, France). Total RNA (2 μg) was reverse-transcribed using High Capacity RNA-to-cDNA™ Kit (Invitrogen) per manufacturer’s instructions. Gene expression levels of PDE1A was quantified using pre-verified Assays-on-Demand TaqMan primer/probe sets (Applied Biosystems, St. Aubin, France) and normalized to 18S ribosomal RNA using the comparative Cycle threshold (Ct) method (2-ΔΔCt).

### Statistical methods

Associations in GWAS were tested by multivariate logistic regression using PLINK software (v 1.0.7, PLINK) [24]. Age and gender were used as covariates. Manhattan plots and QQ-plots were drawn using the R environment (v 2.15.2). Regional plots were drawn by LocusZoom Version 1.1^10^. Meta-analysis was performed by the inverse-variance method. Other statistical methods were the same as those in our previous papers [25, 26].

## SUPPLEMENTARY MATERIALS FIGURES



## Abbreviations

BMPR2: Bone Morphogenetic Protein Receptor Type 2
I/HPAH: idiopathic and heritable pulmonary arterial hypertension
GWAS: genome-wide association study
PAH: pulmonary arterial hypertension
PDE: phosphodiesterase
