# Supplementary material for: A genome-wide association analysis identifies PDE1A|DNAJC10 locus on chromosome 2 associated with idiopathic pulmonary arterial hypertension in a Japanese population
Source: Oncotarget. 2017 Aug 24;8(43):74917–26. doi: 10.18632/oncotarget.20459 (PMC5650389; doi:10.18632/oncotarget.20459)
Supplement: Supplementary file 1 [file oncotarget-08-74917-s001.pdf]

A genome-wide association analysis identifies *PDE1A|DNAJC10* locus on chromosome 2 associated with idiopathic pulmonary arterial hypertension in a Japanese population

SUPPLEMENTARY MATERIALS

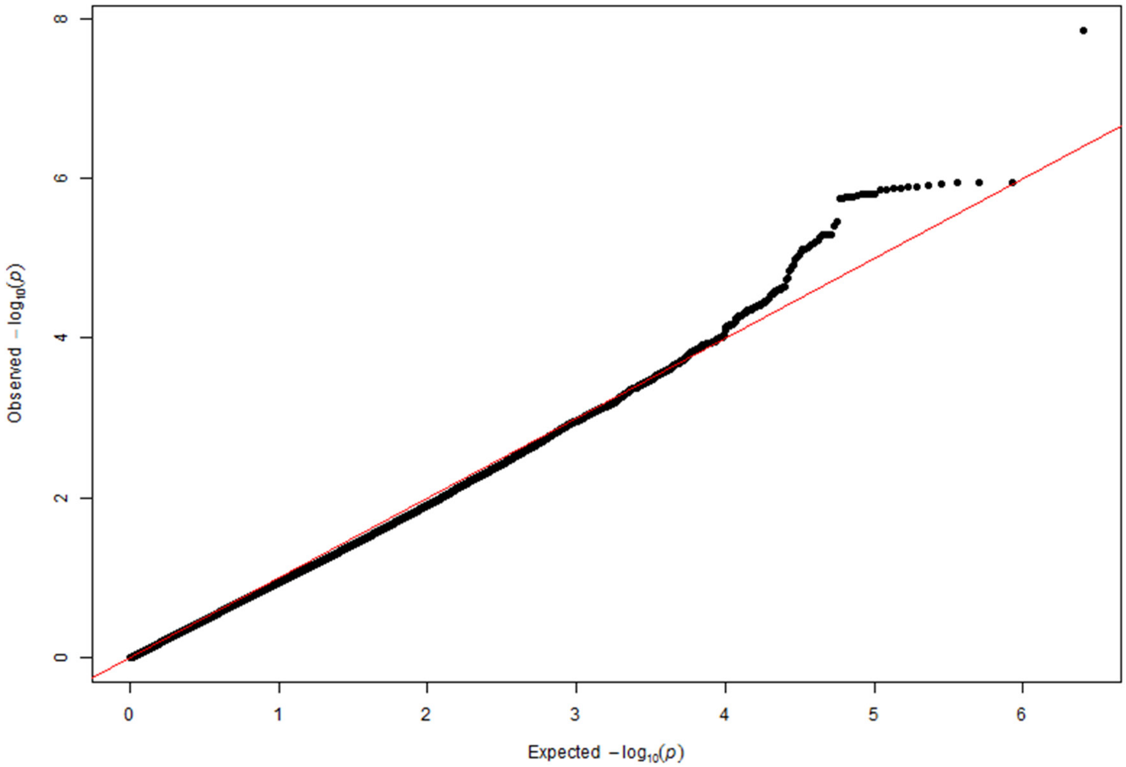

Supplementary Figure 1: QQ-plots of log P-values for the discovery GWAS. Observed – log P-value was compared with expected – log P value.

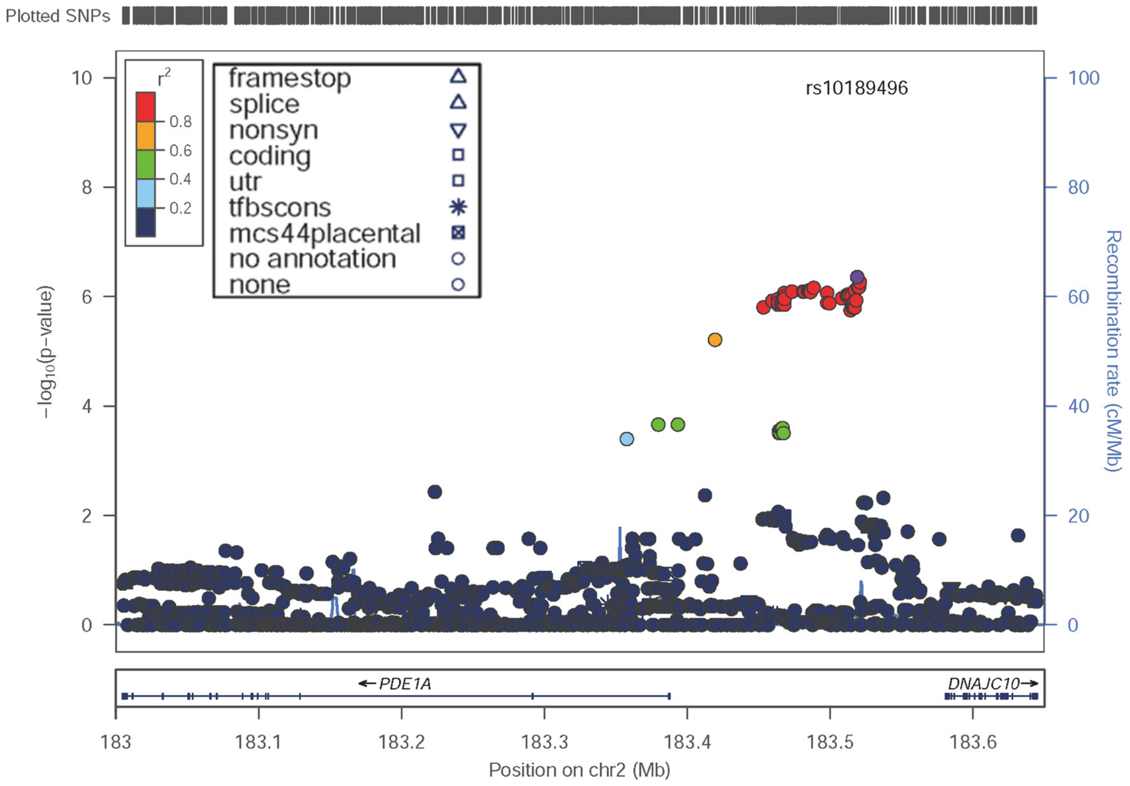

Supplementary Figure 2: Locus-specific plots at the *PDE1A|DNAJC10* locus in the discovery GWAS after imputation. The figure was drawn by LocusZoom Version 1.1[22].

rs #71427857

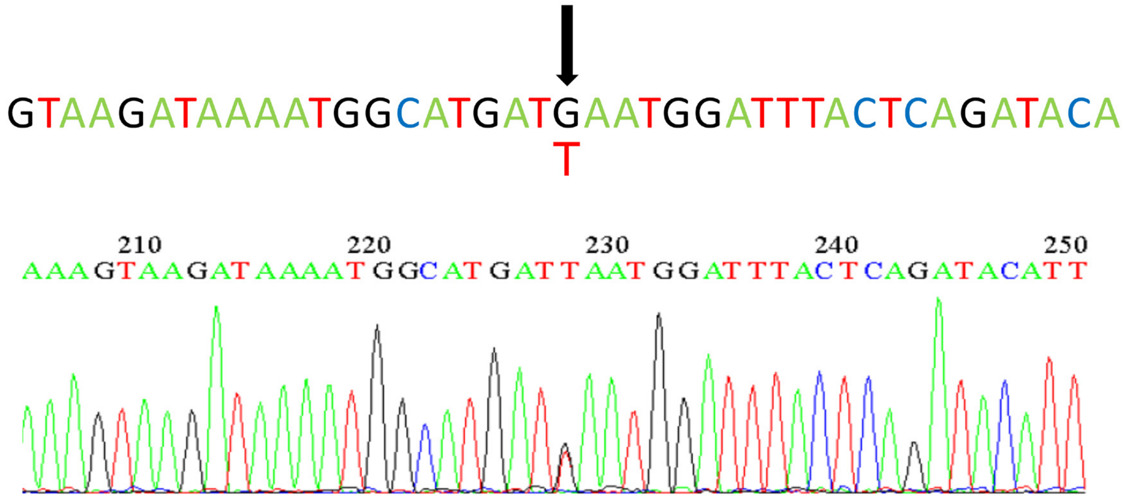

Supplementary Figure 3: Genotyping of minor alleles in rs71427857 by the direct sequence method.
